# Neuromuscular complications after COVID-19 vaccination: a series of eight patients

**DOI:** 10.1007/s13760-022-01941-0

**Published:** 2022-05-02

**Authors:** Wouter Leemans, Sofie Antonis, Wouter De Vooght, Robin Lemmens, Philip Van Damme

**Affiliations:** 1 Department of Neurology, University Hospitals Leuven, Leuven, Belgium; 2 Department of Neurology, Sint-Trudo Hospital, Sint-Truiden, Belgium; 3 Department of Neurosciences, KU Leuven–University of Leuven, Experimental Neurology, and Leuven Brain Institute (LBI), Leuven, Belgium; 4 Center for Brain and Disease Research, Laboratory of Neurobiology, VIB, Leuven, Belgium

**Keywords:** COVID-19, Vaccine, Neuromuscular, CIDP, Guillain-Barré

## Abstract

**Background:**

Several neurologic complications have been reported in close temporal association with both severe acute respiratory syndrome coronavirus-2 (SARS-CoV-2) infection and following vaccination against SARS-CoV-2. Specifically, several cases of Guillain-Barré syndrome (GBS) have been reported in temporal relationship with COVID-19 vaccination, with two small case series describing a specific phenotype with bifacial weakness and paresthesia in the limbs.

**Methods:**

We retrospectively collected patients who developed a new-onset neuromuscular disorder in the first 6 weeks after receiving a COVID-19 vaccine (either first or second dose). The patients were collected from one tertiary care centre and one secondary care centre from February to July 2021.

**Results:**

We report eight patients who developed phenotypically diverse neuromuscular disorders in the weeks following COVID-19 vaccination, with a presumed immune-mediated etiology. In our case series, we report three patients with classical GBS, one patient with bifacial weakness with paresthesia variant of GBS, two patients with subacute-onset chronic inflammatory demyelinating polyneuropathy (CIDP), one patient with brachial plexopathy and one patient with subacute axonal sensorimotor polyneuropathy.

**Conclusions:**

New-onset neuromuscular disorders with onset in the weeks after COVID-19 vaccination can include diverse phenotypes. A causal relationship between these disorders and the vaccine cannot be proven at present, and further epidemiological studies are needed to further investigate this association.

## Introduction

Since the beginning of the severe acute respiratory syndrome coronavirus-2 (SARS-CoV-2) pandemic, several neurologic complications have been described, both as parainfectious (e.g., stroke, viral encephalitis) and postinfectious (e.g., myelitis) phenomena [1]. In addition, neuromuscular complications in close temporal relation with coronavirus disease-2019 (COVID-19) have been reported, the most frequent being Guillain-Barré syndrome (GBS) [2]. Other neuromuscular complications in COVID-19 patients include viral myositis, mononeuritis multiplex and critical illness myopathy [3].

Following the worldwide immunization campaign against SARS-CoV-2, several case reports of GBS in temporal association with the administration of the BNT162b2 (Pfizer-BioNTech), ChAdOx1 nCoV-19 (AstraZeneca) and Ad26.COV2.S (Janssen) COVID-19 vaccines were published. Remarkably, two small case series mentioned GBS patients with a specific combination of bifacial weakness with paraesthesia of limbs (BFP), suggesting that this variant might occur more frequently after COVID-19 vaccination than in ‘classical’ postinfectious GBS [4, 5].

Here, we report eight patients who developed a new-onset neuromuscular disorder after receiving a COVID-19 vaccine. At the time of this study, four vaccines were available in Belgium: BNT162b2 (Pfizer-BioNTech), ChAdOx1 nCoV-19 (AstraZeneca), Ad26.COV2.S (Janssen), and mRNA-1273 (Moderna).

## Methods

We retrospectively collected patients who developed a new-onset neuromuscular disorder in the first 6 weeks after receiving a COVID-19 vaccine (either first or second dose). The patients were collected from University Hospitals Leuven (a tertiary care centre; cases 1–4) and Sint-Trudo Hospital (a secondary care centre; cases 5–8) from February to July 2021. This cohort study was approved by the ethics committee of the University Hospitals Leuven. All patients signed a written informed consent.

## Results

Additional clinical details about the cases are summarized in Table 1.

**Table 1:** Case summaries

	Age (years), gender	Vaccine type	Symptom onset	Clinical findings	Diagnostic test results	Clinical course	Diagnosis
Case 1	79, male	BNT162b2	2 days after first dose	Motor strength: MRC 2/5 LL, 4/5 UL	NCS: subacute demyelinating neuropathy (delayed distal latencies, slow conduction velocity, low amplitude CMAPs with conduction block), prolonged or absent F-waves, absent SNAPs	Treated with IVIg 0.4 g/kg/day for 5 days	Subacute-onset CIDP
				Sensory exam: reduced bilateral LL	CSF: albuminocytological dissociation (WBC 6/µL for protein 110 mg/dL)	Improvement for 2 weeks, then deterioration	
				Reflexes: global areflexia	Serum ganglioside antibodies: GD1a, GD1b, GD2, GD3 and GT1b IgG positive	Retreated with IVIg 3 weeks later	
						Again improvement for 2 weeks, then deterioration	
						Treatment with IVIg at 4 weekly intervals, methylprednisolone and azathioprine was continued	
Case 2	57, male	BNT162b2	4 weeks after second dose	Motor strength: MRC 4/5 for right finger flexion, finger abduction, thumb abduction and –adduction	NCS: lower trunk right brachial plexopathy	Treatment with oral methylprednisolone taper	Brachial plexopathy
				Sensory exam: hypoesthesia medial antebrachial cutaneous nerve	MRI brachial plexus/cervical spine: normal	Mild residual right hand weakness at last clinical follow-up	
				Reflexes: preserved	Serum ganglioside antibodies: negative		
Case 3	80, male	BNT162b2	2 weeks after first dose	Motor strength: normal	NCS: subacute axonal polyneuropathy (low amplitude tibial nerve CMAP with axonal range conduction velocity, slightly prolonged F-waves, absent sural nerve SNAP)	No treatment initiated due to mild complaints	Subacute axonal sensorimotor polyneuropathy
				Sensory exam: reduced sensation to pinprick bilaterally below the knee, vibration sense absent LL and severely reduced UL; positive Romberg sign	Serum antiganglioside antibodies: negative	Stable, mild sensory symptoms in the LL at last clinical follow-up	
				Reflexes: areflexia LL			
Case 4	62, male	ChAdOx1 nCoV-19	4 weeks after first dose	Motor strength: normal	NCS: subacute demyelinating neuropathy (delayed distal latencies, slow conduction velocity, low amplitude CMAPs with partial conduction blocks), prolonged F-waves, absent SNAPs	Treatment with oral methylprednisolone with some effect on sensory complaints (stopped early due to intolerance)	Subacute-onset CIDP
				Sensory exam: reduced sensation to touch in fingers and lower legs, reduced vibration sense bilaterally in LL; positive Romberg sign	Serum ganglioside antibodies: negative	Treatment with IVIg was proposed	
				Reflexes: ankle jerk reflex absent, hyporeflexia for other reflexes			
Case 5	61, female	BNT162b2	2 weeks after first dose	Motor strength: bifacial plegia, LL proximal MRC 2/5, distal 4/5, UL 4/5	NCS: subacute demyelinating polyneuropathy (slow conduction velocity, low amplitude CMAPs and SNAPs, delayed distal latencies, prolonged F-waves)	Treatment with IVIg 0.4 g/kg/day for 5 days	AIDP
				Sensory exam: distal hypesthesia and paresthesias in hands and feet	Serum ganglioside antibodies: anti-sulfatide IgM positive	After initial improvement further worsening with need of enteral feeding	
				Reflexes: global areflexia	CSF: albuminocytological dissociation (WBC 50/µL for protein 1227 mg/L)	Without additional treatment of IVIg or plasmapheresis eventually an excellent recovery with residual mild hypesthesia of her feet and a mild left sided facial palsy	
					Infectious screening negative (Borrelia, Syfilis, HIV, VZV, EBV, CMV, hepatitis B/C/E)		
Case 6	62, male	BNT162b2	11 days after first dose	Motor strength: severe bifacial palsy (House Brackmann V left and VI right). Normal strength in limbs	NCS: subacute demyelinating polyneuropathy (slow conduction velocity, prolonged F-waves)	Treatment with oral methylprednisolone 48 mg/day for 10 days	Variant of GBS with bifacial weakness
				Sensory exam: normal	Serum ganglioside antibodies: anti-GM1 IgG positive	Mild facial weakness on the right side 1 month after onset	
				Reflexes: hyporeflexia LL	CSF: albuminocytological dissociation (WBC 3/µL for protein 1314 mg/L		
Case 7	63, male	ChAdOx1 nCoV-19	1 week after first dose	Motor strength: Hip- and knee flexion MRC 4/5, positive Gowers sign, broad-based gait	NCS: subacute demyelinating polyneuropathy (slow motor conduction velocities, prolonged F-waves, low amplitude SNAPs)	Treatment with IVIg 0.4 g/kg/day for 5 days	AIDP
				Sensory exam: reduced sensation from D10 downward	Serum ganglioside antibodies: negative	Normal strength and gait 1 month after onset	
				Reflexes: preserved	CSF: albuminocytological dissociation (WBC 1/µL for protein 594 mg/L		
					MRI full spine: normal		
Case 8	81, female	BNT162b2	3 weeks after second dose	Motor strength: shoulder abduction right MRC 3/5, other UL 4 + /5. LL proximal 3/5, distal 4/5	NCS: subacute demyelinating polyneuropathy (delayed distal latencies, prolonged F-waves, slow conduction velocities, low amplitude SNAP’s and CMAPs)	Treatment with IVIg 0.4 g/kg/day for 5 days	AIDP
				Sensory exam: ascending hypesthesia and paresthesias with severe sensory ataxia in all 4 limbs	Serum ganglioside antibodies: negative	Residual only mild right shoulder weakness at last clinical follow-up	
				Reflexes: areflexia LL	CSF: albuminocytological dissociation (WBC 1/µL for protein 600 mg/L		

*MRC* medical research council, *LL* lower limbs, *UL* upper limbs, *NCS* nerve conduction studies, *CMAP* compound muscle action potential, *SNAP* sensory nerve action potential, *CSF* cerebrospinal fluid, *WBC* white blood cell count, *IVIg* intravenous immunoglobulins, *CIDP* chronic inflammatory demyelinating polyneuropathy, *MRI* magnetic resonance imaging, *AIDP* acute inflammatory demyelinating polyneuropathy, *GBS* Guillain-Barre syndrome

### Case 1

A 79 year-old man presented with progressive lower extremity weakness starting 2 days after the first dose of the BNT162b2 (Pfizer-BioNTech) COVID-19 vaccine, with a nadir 1 week after the vaccine. Neurological examination showed bilateral weakness in the lower extremities (MRC 2/5) and upper extremities (MRC 4/5), with reduced vibration sense in the lower limbs and global areflexia. Nerve conduction studies showed an inhomogeneous demyelinating sensorimotor neuropathy. Lumbar puncture showed an albumin-cytological dissociation (see Table 1; a virus panel study was not performed). He was treated with intravenous immunoglobulins (IVIG), repeated after 3 weeks due to deterioration after initial improvement. After 2 weeks, the patient deteriorated again, and treatment with IVIG was continued with regular intervals of 4 weeks, while methylprednisolone and azathioprine were associated to his therapy. At last clinical follow-up, muscle strength in the upper extremities remained stable (MRC 4/5), and strength in the lower extremities improved (MRC 3/5 proximally and 4/5 distally). Initially, a diagnosis of Guillain-Barré syndrome was considered. However, since the patient continued to fluctuate more than 8 weeks after onset of the symptoms, a diagnosis of subacute-onset chronic inflammatory demyelinating polyneuropathy (CIDP) was made.

### Case 2

A 57 year-old man presented with right shoulder pain followed within 1 week by motor weakness in the right hand and hypoesthesia of the ulnar side of the right hand and forearm. The complaints started 4 weeks after he received his second dose of the BNT162b2 (Pfizer-BioNTech) COVID-19 vaccine. Clinical examination showed weakness (MRC 4/5) of right finger flexion, finger abduction, thumb abduction and adduction, and hypoesthesia of the medial antebrachial cutaneous nerve territory. EMG and nerve conduction studies showed signs compatible with a right lower trunk brachial plexopathy. MRI of the cervical spine and of the brachial plexus (with and without intravenous gadolinium) were normal. A diagnosis of right lower trunk brachial plexopathy was made. The patient was treated with oral methylprednisolone, with residual mild weakness in the right hand on last clinical follow-up.

### Case 3

An 80 year-old man presented with hypoesthesia in the lower extremities, starting in his feet and gradually ascending to the lower legs, with mild associated pain, but no weakness. The complaints started approximately 2 weeks after he received the first dose of the BNT162b2 (Pfizer-BioNTech) COVID-19 vaccine. Neurological examination showed normal muscle strength, reduced sensation to pinprick bilaterally below the knee, absent vibration sense in the legs and severely reduced vibration sense in the upper extremities. Reflexes were absent in the legs. EMG and nerve conduction studies showed a subacute, axonal sensorimotor polyneuropathy. Lumbar puncture was not performed. No other cause for polyneuropathy could be identified. Due to relatively mild complaints with no motor weakness, no treatment was started. On last clinical follow-up, the symptoms were stable.

### Case 4

A 62 year-old man presented with progressive paresthesia in the extremities, orally and in the genital area, starting 4 weeks after he received the first dose of the ChAdOx1 nCoV-19 (AstraZeneca) vaccine and progressing over several weeks. Furthermore, he complained of unsteadiness while walking. Neurological examination showed normal muscle strength, reduced sensation to touch in the fingertips and lower legs, reduced vibration sense in the legs and a positive Romberg sign. Tendon reflexes were weak with absent ankle jerks. Nerve conduction studies showed a severe demyelinating sensorimotor neuropathy. Initially, a diagnosis of Guillain-Barré syndrome was considered, but given the continued deterioration more than 8 weeks after onset of the complaints, a diagnosis of acute-onset CIDP was made. Lumbar puncture was not performed. An infectious panel (on blood) was negative for Borrelia, EMV, CMV, Hepatitis A, B, C and E. A treatment with oral methylprednisolone was discontinued early due to side effects. Treatment with IV immunoglobulins was proposed, but ultimately refused by the patient due to concern for possible side effects. At last clinical follow-up, the situation was stable.

### Case 5

A 61 year-old woman presented with lower back pain followed by ascending paresthesias in both hand and feet 2 weeks after receiving the first dose of the BNT162b2 (Pfizer-BioNTech) COVID-19 vaccine. Her symptoms quickly evolved to an ascending weakness in the four limbs combined with an extensive bilateral facial weakness and global areflexia. Lumbar puncture showed a cyto-albuminological dissociation with a strongly elevated protein concentration but also a pleocytosis of 50 cells/µl. An extensive infectious screening in serum and cerebrospinal fluid was negative (see Table 1).

Nerve conduction studies showed an inhomogeneous demyelinating sensorimotor polyneuropathy, compatible with acute inflammatory demyelinating polyneuropathy (AIDP). She was treated with IVIG 0.4 g/kg/d for 5 days. After an initial deterioration, she eventually made a very good recovery with only a residual hypesthesia of her feet and a mild left facial palsy.

### Case 6

A 62 year-old man presented with complaints of muscle aches and a severe bilateral facial palsy 11 days after receiving the first dose of the BNT162b2 (Pfizer-BioNTech) COVID-19 vaccine. Neurologic examination showed normal muscle strength with hyporeflexia in the lower extremities.

Neuroborreliosis and neurosarcoidosis were ruled out as possible alternative diagnoses by laboratory testing and chest CT. MRI of his cranial nerves revealed no structural abnormalities.

Nerve conduction studies demonstrated signs of mild demyelination of the motor nerves in both arms and legs.

We established the diagnosis of a rare variant of the Guillain-Barre syndrome with only bifacial weakness. He received a 10 day course of 48 mg of methylprednisolone a day. He made a good recovery with only a mild right facial weakness 1 month after onset.

### Case 7

A 63 year-old man presented with subacute development of proximal weakness in his legs starting 1 week after the first dose of the ChAdOx1 nCoV-19 (AstraZeneca) vaccine. Neurological examination demonstrated reduced motor strength of predominantly hip and knee flexors with a positive Gowers sign and broad-based gait. In a second phase, he also experienced a band-like hypesthesia from D10 downward with neuropathic pains in his lower limbs. Given the initial presentation of proximal muscle weakness without sensory symptoms, a myopathy was considered. However, nerve conduction studies showed a demyelinating sensorimotor polyneuropathy, compatible with AIDP, and needle EMG showed no signs of a myogenic disorder. Due to the clear sensory level that developed, we also considered a myelopathy (e.g. myelitis), however, MRI full spine revealed no abnormalities and CSF showed a cyto-albuminological dissociation but no elevated white cell count.

Given the test results, a diagnosis of Guillain-Barré syndrome was made.

He was treated with IVIG with a dose of 0.4 g/kg/d for 5 days and made an excellent recovery with a normal strength and gait 1 month later.

### Case 8

An 81 year-old woman, with a complex medical history of a malignant melanoma and peritoneal metastases of a colon carcinoma, presented with ascending hypesthesia and paresthesias in her legs and hands. She underwent an experimental pressurized intraperitoneal aerosol chemotherapy (PIPAC) treatment with oxaliplatin 2 weeks earlier and received her second dose of the BNT162b2 (Pfizer-BioNTech) COVID-19 vaccine approximately 3 weeks earlier.

She experienced rapidly ascending motor weakness in her legs and right arm and had a very pronounced sensory ataxic gait. She also had autonomic failure with cardiac arrhythmias and urinary retention. MRI of the brain and spinal cord were normal. Paraneoplastic antibodies in serum were negative and cytologic examination of cerebrospinal fluid showed no malignant cells; there was, however, a cyto-albuminological dissociation (WBC 1/µL for protein 600 mg/L). Nerve conduction studies were compatible with AIDP. In the differential diagnosis, we considered a classical Guillain-Barré syndrome (possibly related to recent vaccination) or an acute neuropathy related to recent treatment with oxaliplatin. She received IVIG with a dose of 0.4 g/kg/d for 5 days. She only had residual mild right shoulder weakness at last clinical follow-up.

## Discussion

In this paper, we report eight patients who developed a neuromuscular disorder within 1–4 weeks after receiving the first or second dose of COVID-19 vaccination. Our patients had diverse phenotypes, including classical GBS, bifacial weakness variant of GBS, (sub) acute-onset CIDP, brachial plexopathy, and subacute axonal polyneuropathy. While several cases of GBS after COVID-19 vaccination have already been reported, including series of the bifacial weakness variant [4, 5], to the best of our knowledge, this is the first report of CIDP or brachial plexopathy occurring after COVID-19 vaccination.

A causal relationship between vaccination and GBS has been suspected since the 1976 ‘swine flu’ pandemic in the United States of America, when during a mass vaccination campaign, a spike in GBS incidence occurred [6]. Since then, case reports occurring shortly after administration of several vaccines have been reported in the literature, without proven causality [6]. A recent nested case–control study in China found no evidence of an increased risk of GBS after vaccination in general, nor after influenza vaccination specifically [7].

For CIDP, an association with antecedent infection (or vaccination) has been less clear than for GBS. A recent study showed 9.3% of CIDP patients reported an antecedent infection and 1.1% reported receiving an influenza vaccination in the 6 weeks prior to CIDP onset [8]. These patients were more likely to suffer from acute-onset CIDP, which is also reflected in our series. However, as in GBS, a causal relationship between vaccination and CIDP is not proven [8].

In Belgium, 7.775.552 first doses of a COVID-19 vaccine were administered between February and July 2021, as well as 6.570.466 second doses [9]. The incidence of neuromuscular complications following COVID-19 vaccine administration cannot be determined based on our data, however, since only patients from two hospitals were included. To our knowledge, no epidemiological data concerning neuromuscular complications after COVID-19 vaccination have been published to date.

In conclusion, we report the cases of eight patients with phenotypically diverse neuromuscular complications occurring after COVID-19 vaccination. To our knowledge, no other cases of CIDP or brachial plexopathy following COVID-19 vaccination have been reported so far. We also report another case of the bifacial weakness with paraesthesia variant of GBS, of which some small case series have already been reported. These various presentations strengthen the association between COVID-19 vaccination and particular GBS phenotypes. While a causal relationship between these disorders and the vaccine cannot be proven at present, the temporal association is remarkable. Further epidemiological studies are needed to further investigate the association between COVID-19 vaccination and peripheral nerve disorders.
